# Structural basis of glucosinolate recognition and polyspecific transport by the glucosinolate transporter GTR1

**DOI:** 10.1016/j.jbc.2026.113190

**Published:** 2026-05-24

**Authors:** Jing Nan, Hao Xu, Sisi Kang, Guohui Xie, Jun Zhao, Sisi Li

**Affiliations:** 1 International Cancer Center, Guangdong Key Laboratory of Genome Instability and Human Disease Prevention, Department of Biochemistry and Molecular Biology, Shenzhen University Medical School, Shenzhen, Guangdong, China; 2 State Key Laboratory of Wheat Improvement, Peking University Institute of Advanced Agricultural Sciences, Shandong Laboratory of Advanced Agricultural Sciences in Weifang, Weifang, Shandong, China; 3 Shenzhen Key Laboratory of Plant Genetic Engineering and Molecular Design, Institute of Plant and Food Science and Institute for Biological Electron Microscopy, Department of Biology, School of Life Sciences, Southern University of Science and Technology, Shenzhen, Guangdong, China

**Keywords:** glucosinolate transporter, GTR1, cryo-EM, substrate recognition, NPF transporter

## Abstract

Glucosinolates (GLSs) are sulfur-rich secondary metabolites characteristic of *Brassicales* and play central roles in plant defense. While the biosynthesis and long-distance allocation of GLSs have been extensively studied, the molecular principles underlying their membrane transport by Glucosinolate Transporters (GTRs) remain poorly understood. Here, we report cryogenic electron microscopy (cryo-EM) structures of *Arabidopsis thaliana* GTR1 (AtGTR1) in apo and substrate-bound states. Structures of AtGTR1 in complex with the glucobrassicin (3-indolylmethyl glucosinolate, I3M), were determined at two pH conditions, capturing the transporter in a conserved inward-open conformation. The structures reveal a central substrate-binding cavity formed at the interface of the N- and C-terminal domains. Structural analyses uncover a conserved recognition strategy in which AtGTR1 selectively engages the common glucosinolate scaffold while tolerating substantial variation in side chains, thereby explaining its polyspecificity. These findings establish a structural framework for GTRs and provide mechanistic insights that enable rational manipulation of GLSs allocation for crop improvement.

Oilseed rape (*Brassica napus*) is one of the most important oilseed crops worldwide, serving as a major source of edible vegetable oil and protein-rich animal feed (1). However, the economic value of rapeseed meal, a byproduct of oil extraction, is substantially reduced by the accumulation of glucosinolates (GLSs), a class of sulfur-rich secondary metabolites (2, 3). While GLSs constitute a hallmark chemical defense of *Brassicales* plants and play essential roles in protecting plants against herbivores and pathogens in natural environments (4), they act as potent antinutritional and toxic compounds in agricultural settings, particularly for livestock consumption. Decades of breeding efforts aimed at reducing seed GLS content have encountered a fundamental constraint known as the “double-low” dilemma: lowering GLS levels in seeds is often accompanied by a concomitant loss of GLS-based defense in vegetative tissues, leading to increased pest susceptibility and reduced crop resilience (5, 6). As a result, the challenge of uncoupling seed quality from vegetative defense has emerged as a long-standing and unresolved problem in *Brassica* crop improvement (7).

A unique physiological feature of GLS allocation provides a potential solution to this dilemma. Unlike primary metabolites that are synthesized ubiquitously, GLS accumulation in seeds does not rely on *de novo* biosynthesis within the embryo. Instead, seeds function as metabolic sinks that depend almost entirely on long-distance transport of GLS synthesized in source tissue, such as mature leaves and silique walls (8, 9, 10). This spatial separation between biosynthesis and storage creates a critical opportunity: selective interference with GLS transport into seeds could, in principle, eliminate seed toxicity while preserving defensive capacity in vegetative organs (11). This concept has given rise to the strategy of “transport engineering”, which aims to manipulate the transporter cascade responsible for GLS source-to-sink mobilization (12, 13, 14, 15).

Genetic and physiological studies in *Arabidopsis thaliana* have elucidated the basic architecture of this transport pathway. GLS translocation requires a coordinated relay across multiple membrane barriers, involving UMAMIT transporters that mediate GLS efflux into the apoplast and glucosinolate transporters (GTRs) that catalyze high-affinity, proton-coupled re-uptake for phloem loading and seed provisioning (16, 17, 18, 19, 20, 21). GTRs are responsible for the cellular import of GLSs from the apoplastic space into the cytoplasm. The essential role of GTRs is underscored by the *gtr1gtr2* double mutant, which produces seeds almost completely devoid of GLS (11). In addition to their well-established role at the plasma membrane, emerging evidence suggests that GTRs may also function in extracellular vesicles as defense system (22). Importantly, targeted genome editing of GTR homologs in *Brassica* crops has demonstrated that this mechanism is conserved and agriculturally exploitable, enabling the generation of varieties with low seed GLS content while maintaining robust leaf defense and yield (23).

Despite these advances, the rational design of GTR-based transport engineering remains severely constrained by a lack of mechanistic understanding at the molecular level. GTRs belong to the Nitrate/Peptide Transporter Family (NPF), a large group of proton-coupled transporters whose structural characterization is largely limited to the nitrate transporter AtNRT1.1 (17, 24, 25, 26). Although AtNRT1.1 provides a valuable architectural framework, it is optimized for the transport of nitrate, a small inorganic anion, and therefore offers limited insight into how GTRs accommodate GLSs, a group of large, amphipathic molecules with bulky and chemically diverse side chains (27). Moreover, GTRs exhibit pronounced substrate promiscuity, transporting not only structurally distinct GLSs but also phytohormones such as gibberellin (GA) and jasmonic acid (JA), suggesting a mechanism of polyspecific substrate recognition that remains structurally undefined (28, 29, 30, 31).

Here, we determined cryogenic electron microscopy (cryo-EM) structures of the glucosinolate transporter GTR1 in both the apo state and in complex with I3M. These structures uncover a conserved recognition strategy in which GTR1 selectively engages the common glucosinolate core while tolerating substantial variation in side chains, thereby explaining its polyspecific transport capability. Together, our findings establish an atomic framework for glucosinolate transport by GTRs, extend current understanding of substrate recognition in the NPF transporter family, and provide a conceptual basis for engineering glucosinolate allocation in crops.

## Results

### Structure determination of AtGTR1

**Figure 1:**
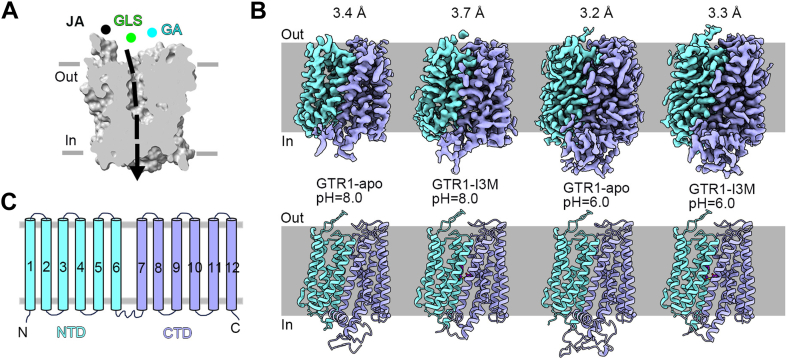
**Structural characterization of AtGTR1 under different conditions.***A*, schematic diagram illustrating the biological functions of AtGTR1 in *Arabidopsis thaliana*. *B*, cryo-EM reconstructions and corresponding atomic models of AtGTR1. The *upper panel* displays the cryo-EM density maps, while the *lower panel* shows the overall ribbon structures of AtGTR1 in the apo and I3M-bound states at pH 8.0 and pH 6.0, respectively. *C*, topology diagram of AtGTR1. The N-terminal domain (NTD) and C-terminal domain (CTD) are colored in *cyan* and *magenta*, respectively.

To establish a structural framework for understanding substrate recognition and transport by GTRs, we set out to determine the structure of *A. thaliana* GTR1 (AtGTR1), a multifunctional member of the NPF family known to mediate the transport of GLSs as well as GA and JA (Fig. 1*A*). AtGTR1 was heterologously expressed in Expi293E cells and purified to homogeneity, yielding a monodisperse protein preparation suitable for high-resolution cryo-EM analysis (Fig. S1). To probe the structural properties of AtGTR1 under conditions relevant to its proton-coupled transport activity, we determined cryo-EM structures of AtGTR1 in both ligand-free and substrate-bound states at two distinct pH conditions. Specifically, structures were solved for apo AtGTR1 and for AtGTR1 in complex with I3M at pH 8.0 and pH 6.0, corresponding to putative cytosolic and vesicular environments, respectively. These datasets yielded reconstructions at resolutions ranging from 3.2 to 3.7 Å (Figs. 1*B* and S2–S5), enabling unambiguous model building of the transmembrane domain.

Across all four structures, AtGTR1 adopts a conserved architecture comprising twelve transmembrane α-helices (TMs) organized in the canonical major facilitator superfamily (MFS) fold, with both N- and C-termini located on the cytoplasmic side of the membrane (Fig. 1*C*). Notably, comparison of the structures obtained under different pH conditions and ligand-binding states revealed no substantial global conformational rearrangements; instead, all structures converge on a highly similar inward-open conformation (Fig. S6). Together, these structures define a stable inward-open conformation of AtGTR1 that is largely insensitive to pH variation and substrate binding, providing a unified structural framework for analyzing GLS recognition and transport.

### Overall architecture of AtGTR1 reveals a central substrate-binding cavity

**Figure 2:**
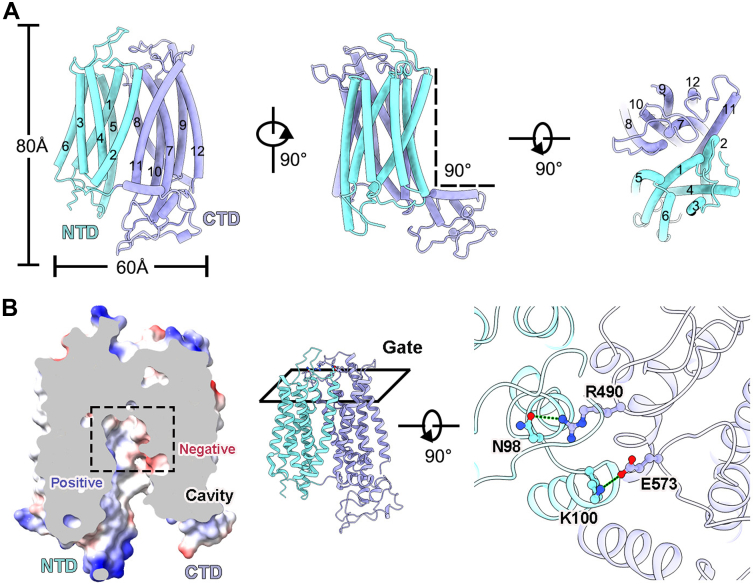
**Overall architecture and substrate-binding cavity of apo AtGTR1 at pH 6.0.***A*, ribbon representation of the apo AtGTR1 structure, showing an inward-open conformation. *B*, detailed view of the central cavity and the extracellular gate. The molecular surface or residues defining the cavity are shown to illustrate the transport path environment.

To define the structural basis for substrate binding and translocation, we next examined the overall architecture of AtGTR1. Of the 636 residues in AtGTR1, 547 are well resolved in the cryo-EM maps, with unresolved regions largely confined to the flexible N- and C-terminal segments (residues 1–59 and 607–636) (Fig. 2*A*). The resolved structure is composed of twelve transmembrane helices organized into two pseudosymmetric bundles: an N-terminal domain (NTD; TMs 1–6) and a C-terminal domain (CTD; TMs 7–12), separated by a prominent intracellular loop connecting TM6 and TM7.

This intracellular domain forms a rigid, helix-rich structure that intersects the transmembrane domain at an approximately perpendicular angle and partially spans the transport pathway (Fig. 2*A*). The surface of this domain facing the membrane is enriched in hydrophobic residues (Fig. S7*A*), suggesting stable peripheral association with the lipid bilayer and a potential role in stabilizing the inward-open conformation. Together, the NTD and CTD converge to form a central cavity at their interface, which constitutes the primary substrate-binding site of AtGTR1 (Fig. 2*B*). Notably, this cavity exhibits a pronounced electrostatic asymmetry, with a positively charged surface contributed by the NTD and a negatively charged surface formed by the CTD.

Consistent with the inward-open state observed across all structures, the extracellular gate of AtGTR1 is tightly sealed by a network of hydrogen bonds and salt bridges, including interactions between N98 and R490 as well as K100 and E573 (Fig. 2*B*). N98 further engages in stabilizing interactions with residues in the TM3-TM4 loop, which is reinforced by an intramolecular disulfide bond between C164 and C170. This disulfide linkage, not previously observed in other POT/NPF family members (32, 33), likely contributes to gate stabilization and structural rigidity at the extracellular entrance. (Fig. S7*B*). Mutations of C164 and C170 to alanine resulted in the reduction of transport activity compared with the wild-type transporter (Fig. S7*C*).

In summary, the architecture of AtGTR1 defines a rigid inward-open scaffold featuring an electrostatically partitioned central cavity and a tightly sealed extracellular gate, providing the structural framework for selective GLS recognition and transport.

### Substrate binding sites of AtGTR1 in the inward-open state

**Figure 3:**
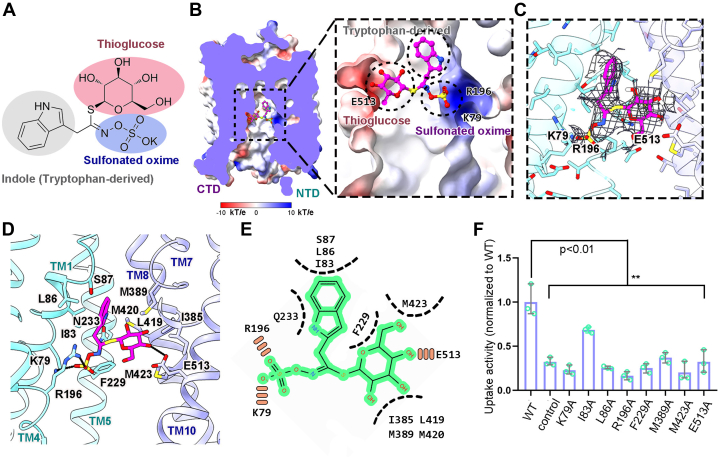
**Substrate binding site and transport mechanism of AtGTR1.***A*, chemical structures of I3M. *B*, Slab view of the electrostatic surface of I3M binding pocket. *C*, Cryo-EM density of the bound I3M. The map is shown as a *gray mesh* contoured at 5σ. *D*, Close-up view of the substrate-binding site. AtGTR1 is shown in a ribbon representation, illustrating the I3M-binding mode within the inward-facing (lumen-facing) pocket. *E*, detailed interaction network between AtGTR1 and I3M. Hydrophobic interactions and polar contacts are indicated by *black* and *orange dashed lines*, respectively. *F*, Functional validation of AtGTR1 transport activity. Uptake levels are normalized to the wild-type (WT) transporter. Data are presented as mean ± SD from n = 3 independent biological replicates. Statistical significance was determined by one-way ANOVA followed by Tukey’s multiple comparisons test (∗∗*p* < 0.01). Individual data points are overlaid as *green dots*.

To elucidate how AtGTR1 selectively recognizes GLS, we next analyzed the substrate-binding site using structures of AtGTR1 in complex with the 3-indolylmethyl glucosinolate (I3M), the predominant tryptophan-derived GLS in *Arabidopsis* seeds (Fig. 3*A*). Cryo-EM structures of the AtGTR1-I3M complex were determined at pH 8.0 and pH 6.0 to assess potential pH-dependent effects on substrate binding, yielding reconstructions at resolutions of 3.7 Å and 3.2 Å, respectively. In both conditions, AtGTR1 remains in an inward-open conformation, with minimal structural deviation from the apo state (RMSD of 0.50 Å), and I3M occupies an identical binding site within the central cavity (Fig. S6, *C* and *D*).

A well-defined electron density was observed within the central cavity, allowing unambiguous placement of I3M (Fig. 3, *B* and *C*). The binding pocket is formed at the interface between the NTD and CTD and exhibits a striking electrostatic and chemical partitioning: a positively charged region contributed by the NTD, a negatively charged patch from the CTD, and an intervening hydrophobic surface (Fig. 3*B*). This tripartite architecture is highly complementary to the conserved chemical features of GLS, which comprise a negatively charged sulfonated oxime, a thioglucose core, and a variable hydrophobic side chain.

Consistent with this complementarity, the sulfonated oxime group of I3M is positioned within the positively charged region, where it forms hydrogen-bonding and salt-bridge interactions with R196 and K79 (Fig. 3, *D* and *E*). The thioglucose moiety extends toward the negatively charged region and engages in a hydrogen bond with E513, while simultaneously forming hydrophobic contacts with residues F229, I385, M389, L419, M420, and M423, stabilizing the core of the ligand. The indole side chain of I3M is accommodated within the hydrophobic portion of the cavity and is primarily anchored by interactions with L86, I83, S87, and Q233 (Fig. 3, *D* and *E*). Notably, in addition to the canonical MFS helices involved in substrate binding (TMs 1, 4, 7, and 10), AtGTR1 recruits TM5 and TM8 to engage I3M, thereby expanding the binding surface and shaping a pocket capable of accommodating bulky and chemically diverse substrates (Fig. 3*D*). To validate the functional relevance of these structural observations, we assessed I3M transport activity using a HEK293T cell-based uptake assay. Mutations of residues involved in coordinating the sulfonated oxime, thioglucose core, or indole side chain of I3M resulted in a marked reduction of transport activity compared with the wild-type transporter (Fig. 3*F*), confirming their essential roles in substrate recognition and binding.

To further examine how GTR1 accommodates structurally diverse glucosinolates, we performed *in silico* docking analyses using representative substrates with distinct side chains, including the aliphatic glucosinolate glucoerucin (4MTB) and the benzenic glucosinolate glucosinalbate (Fig. S8). In both cases, the conserved glucosinolate core adopts a binding mode similar to that of I3M, while the variable side chains extend into a relatively spacious region of the pocket without apparent steric clashes (Fig. S8). Together, these results reveal that AtGTR1 recognizes GLS through an electrostatically and chemically partitioned binding cavity that precisely accommodates the conserved GLS core while tolerating diverse side chains, providing a structural basis for both specificity and polyspecificity in GTR-mediated transport.

### Structural comparisons of AtGTR1 with other NPF transporters

To gain mechanistic insight into the transport cycle of AtGTR1 beyond the inward-open conformation captured here, we performed structural comparisons with representative members of the NPF family. Structural superposition of inward-open AtGTR1 with *Arabidopsis* NRT1.1 (25), the only plant NPF transporter whose structure has been resolved in a comparable state, revealed a close overall similarity, with an RMSD of 1.48 Å across the transmembrane domain (Fig. S6*E*). This conservation suggests that AtGTR1 and AtNRT1.1 share a common inward-open architecture despite their distinct substrate specificities.

**Figure 4:**
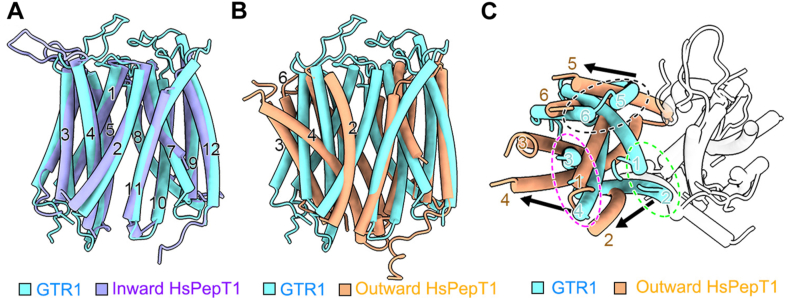
**Structural comparison and proposed transport mechanism of AtGTR1.***A*, structural superimposition of inward-open AtGTR1 (apo state at pH 6.0, this study) and inward-open *Homo sapiens* PepT1 (HsPepT1; PDB ID: 7S8U). *B*, structural comparison between inward-open AtGTR1 (this study) and outward-open HsPepT1 (PDB ID: 7PN1). *C*, conformational transition analysis. Superimposition of AtGTR1 (inward-open) and HsPepT1 (outward-open) based on their C-terminal domains (CTDs) highlights the significant rigid-body movement and conformational rearrangement of the N-terminal domain (NTD).

During the preparation of this manuscript, a preprint and a related work reported AtGTR1 structures in both outward-open and inward-open conformations (34, 35). The overall architecture and substrate-binding mode are largely consistent with our structures. These complementary studies together provide a more complete framework for understanding the GTR1 transport cycle. To further place our structure into a broader mechanistic framework, we compared AtGTR1 with the human peptide transporter PepT1 (HsPepT1), a structurally characterized member of the NPF/POT superfamily with both inward-open and outward-open conformations available (36, 37). Despite their relatively low sequence identity (∼25%) (Fig. S9), superposition of inward-open AtGTR1 with inward-open HsPepT1 yielded an RMSD of 1.84 Å within the transmembrane domain, indicating a highly conserved inward-facing conformation across plant and animal NPF/POT family members (Fig. 4*A*). In contrast, alignment of inward-open AtGTR1 with outward-open HsPepT1 resulted in a substantially larger RMSD of 5.04 Å (Fig. 4*B*), reflecting pronounced conformational differences associated with extracellular gate opening.

Detailed comparison revealed that the transition between inward- and outward-open states is dominated by large-scale rearrangements of the NTD. When the structures were aligned based on the CTD, all six NTD TMs exhibited coordinated shifts between the two conformations (Fig. 4*C*). In the outward-open state, TM1 and TM2 undergo a pronounced tilt of approximately 45°, leading to dilation of the extracellular gate, whereas TM3-TM6 display more modest tilting motions ranging from ∼10° to ∼20°. In contrast, the CTD remains comparatively rigid, suggesting that it serves as a structural anchor during the transport cycle (Fig. 4*C*). In this model, outward movement of the NTD TMs opens the extracellular gate to permit substrate loading from the apoplast. Subsequent substrate binding stabilizes a conformational transition in which the NTD shifts inward, sealing the extracellular gate and adopting an inward-open state that allows release of the glucosinolate into the cytosol. This alternating-access mechanism is consistent with conserved NPF transporter dynamics while accommodating the unique chemical properties of GLS.

## Discussion

In this study, we determined the cryo-EM structures of AtGTR1 in both apo and GLS-bound states, capturing the transporter exclusively in an inward-open conformation. Although AtGTR1 has previously been reported to form dimers at the plasma membrane based on bimolecular fluorescence complementation assays, and cooperative dimerization has also been described for the related transporter AtNRT1.1 (38, 39, 40), our biochemical and structural analyses consistently indicate a monomeric organization of AtGTR1 under the conditions examined here. AtGTR1 eluted as a single, monodisperse peak during size-exclusion chromatography, and all four resolved structures reveal a monomeric architecture. While these observations do not exclude the possibility of transient or context-dependent oligomerization *in vivo*, they suggest that a single AtGTR1 protomer is sufficient to support substrate recognition and transport.

At the molecular level, our structures reveal that AtGTR1 recognizes GLS through a conserved yet asymmetric binding strategy. The substrate-binding cavity is organized to tightly engage the invariant sulfonated oxime–thioglucose core *via* defined electrostatic and hydrogen-bonding interactions, while accommodating chemically diverse side chains within a largely permissive hydrophobic environment. This separation between a constrained core-recognition region and a flexible side-chain pocket provides a structural explanation for how GTR1 maintains specificity for the GLS scaffold while tolerating substantial variation among different GLS species (Fig. S8). Although this recognition mode is clearly supported by the GLS-bound structures presented here and sequence alignment showed the ligand-binding residues of GTRs are conserved (Fig. S10), whether similar principles operate more broadly across the NPF transporter family remains an open question. NPF members transport chemically diverse substrates, including nitrate, peptides, and phytohormones, yet direct structural evidence for shared mechanisms of polyspecificity is currently limited. In this context, the reported ability of AtGTR1 to transport phytohormones such as GA and JA that lack the canonical GLS core (Fig. S8) cannot be explained by the binding mode observed in our structures. These findings suggest that GTR1 may employ additional binding configurations or conformational states for distinct substrate classes, an idea that will require direct structural and functional validation in future studies.

Nevertheless, the structural insights presented here establish a foundation for understanding GLS transport at the atomic level. By revealing how GTR1 recognizes conserved chemical features of GLS while accommodating side-chain diversity, this work provides a mechanistic framework for rational transport engineering. Such knowledge has direct implications for crop improvement strategies, highlighting the broader agricultural and biological significance of GTR-mediated transport.

## Experimental procedures

### Expression and purification of AtGTR1

The cDNA fragment encoding *A. thaliana GTR1* (UniProt ID: Q944G5) was amplified and cloned into a pCAG vector, incorporating a C-terminal His-tag and a Flag-tag. AtGTR1 was expressed in Expi293E cells cultured in SMM 293T-II medium at 37 °C with 5% CO_2_. Transfection was initiated at a cell density of approximately 2.0 × 10^6^ cells/ml. For 1 L of culture, 2 mg of plasmid DNA was pre-incubated with 4 mg of polyethylenimine (PEI) in 50 ml of fresh medium for 15 min at room temperature before addition to the cells. Cells were harvested 48 h post-transfection and resuspended in buffer A (150 mM NaCl, 25 mM HEPES, pH 7.4). The membrane fraction was solubilized in buffer A supplemented with 0.5% (w/v) n-dodecyl-β-D-maltopyranoside (DDM, Anatrace) and 0.05% (w/v) cholesteryl hemisuccinate (CHS) for 2 h at 4 °C. After centrifugation at 18,000 rpm for 1 h, the supernatant was incubated with anti-Flag M2 affinity resin (GenScript). The resin was washed with buffer B (150 mM NaCl, 25 mM HEPES, pH 7.4, 0.01% LMNG, and 0.001% CHS). The protein was further cleaned using Ni-NTA affinity chromatography (Cytiva). Finally, size-exclusion chromatography was performed using a Superose 6 Increase 10/300 Gl column (Cytiva). For pH 8.0 samples, the column was equilibrated in 150 mM NaCl, 25 mM Tris (pH 8.0), 0.005% LMNG, and 0.0005% CHS. For pH 6.0 samples, the buffer was replaced with 150 mM NaCl, 25 mM Bis-Tris (pH 6.0), 0.005% LMNG, and 0.0005% CHS.

### Cryo-EM sample preparation and data acquisition

Purified AtGTR1 was concentrated to approximately 8 mg/ml. For the substrate-bound state, 10 mM indolyl-3-methyl glucosinolate (I3M) was added to the protein solution. Aliquots (3 μl) of the protein sample (with or without I3M) were applied to glow-discharged Quantifoil R1.2/1.3 300-mesh gold holey carbon grids. The grids were blotted for 4 s at 4 °C with 100% humidity and vitrified by plunging into liquid ethane using a Vitrobot Mark IV (Thermo Fisher Scientific).

Data collection was performed on a Titan Krios G3i microscope (Thermo Fisher Scientific) operated at 300 kV, equipped with a K3 Summit direct electron detector (Gatan) and a Selectris X energy filter at the PKU-IAAS Cryo-EM Center. Automated data acquisition was controlled *via* EPU software at a nominal magnification of 105000×, corresponding to a calibrated pixel size of 0.855 Å. Movie stacks were recorded in counting mode with a total dose of ∼60 e^–^/A^2^ distributed over 40 frames. The defocus range was set from −1.0 μm to −2.0 μm. A total of 5218 (apo, pH 8.0), 4907 (I3M-bound, pH 8.0), 5873 (apo, pH 6.0), and 7983 (I3M-bound, pH 6.0) movie stacks were collected.

### Cryo-EM data processing and structure determination

Beam-induced motion correction and Contrast Transfer Function (CTF) estimation were performed using cryoSPARC (v3.3.2) (41). For the apo AtGTR1 (pH 8.0) dataset, particles were picked in blob and extracted binned by two, yielding 5,261,707 initial particles. Multiple rounds of 2D classification were performed to remove low-quality particles, yielding 645,775 selected particles. These particles were re-extracted by bin1 and subjected to *ab initio* refinement, generating an intermediate initial model at 3.95 Å from 48,257 particles. This map was then used as a "seed" for seed-facilitated 3D classification, which significantly expanded the high-quality particle pool to 295,415 particles. Subsequent *ab initio* reconstruction and 3D classification further sorted the particles, retaining 151,702 particles. Of these, 91.8% corresponding to a homogeneous, well-resolved conformational state were selected for non-uniform and local refinement. Finally, a high-quality density map with an overall resolution of 3.43 Å was obtained from 139,209 particles. Similar processing pipelines were applied to the other three datasets. For the AtGTR1-I3M complex (pH 8.0), a 3.66 Å map was generated from 20,469 particles; for AtGTR1-apo (pH 6.0), a final 3.20 Å map was obtained from 172,172 particles; and for the AtGTR1-I3M-bound state (pH 6.0), a 3.30 Å map was reconstructed from 61,566 particles. An AlphaFold2-predicted model of AtGTR1 served as the initial template (42). Atomic models were manually adjusted in Coot (43) and refined using the real-space refinement module in PHENIX (44). The quality of the final models was validated using MolProbity within the PHENIX package (43, 44, 45). This pH 8.0 apo structure was utilized as the starting model for building the remaining structures. Structural statistics are summarized in Table S1. All structural figures were prepared using UCSF ChimeraX (46).

### *In vitro* transport activity assays

The transport activity of AtGTR1 was evaluated by monitoring the uptake of I3M in HEK293T cells. Cells were seeded in 24-well plates and transfected with 0.5 μg of DNA per well. After 36 h, cells were washed and incubated in an uptake buffer (150 mM NaCl, 20 mM MES, pH 5.8). The transport reaction was initiated by adding 100 μM I3M and incubated at 37 °C for 1 h. The reaction was quenched by washing twice with ice-cold uptake buffer. Intracellular metabolites were extracted with 70% methanol at 20 °C for 1 h. The supernatant was analyzed by high-performance liquid chromatography (HPLC). Statistical significance was determined by one-way ANOVA followed by Tukey’s multiple comparisons test using GraphPad Prism 8.0.1.

## Data availability

Atomic coordinates have been deposited in the Protein Data Bank (http://www.rcsb.org) under the accession codes 23DA, 23CZ, 23CN, and 23DB, respectively. The corresponding electron microscopy maps have been deposited in the Electron Microscopy Data Bank (https://www.ebi.ac.uk/pdbe/emdb/), under the accession codes EMD-68876, EMD-68875, EMD-68863, and EMD-68877, respectively.

## Supporting information

This article contains supporting information.

## Conflict of interest

The authors declare the following financial interests/personal relationships which may be considered as potential competing interests:

I, Sisi Li, confirm that the manuscript has been read and approved by all named authors and that there are no other persons who satisfied the criteria for authorship but are not listed. I further confirm that I have been given the authority by all co-authors to sign this declaration on their behalf.
