# Supplementary material for: Structural basis of glucosinolate recognition and polyspecific transport by the glucosinolate transporter GTR1
Source: J Biol Chem. 2026 May 24;302(7):113190. doi: 10.1016/j.jbc.2026.113190 (PMC13311161; doi:10.1016/j.jbc.2026.113190)
Supplement: Supporting Figures and Table [file mmc1.docx]

Supplementary Information

**Supplementary Figures**


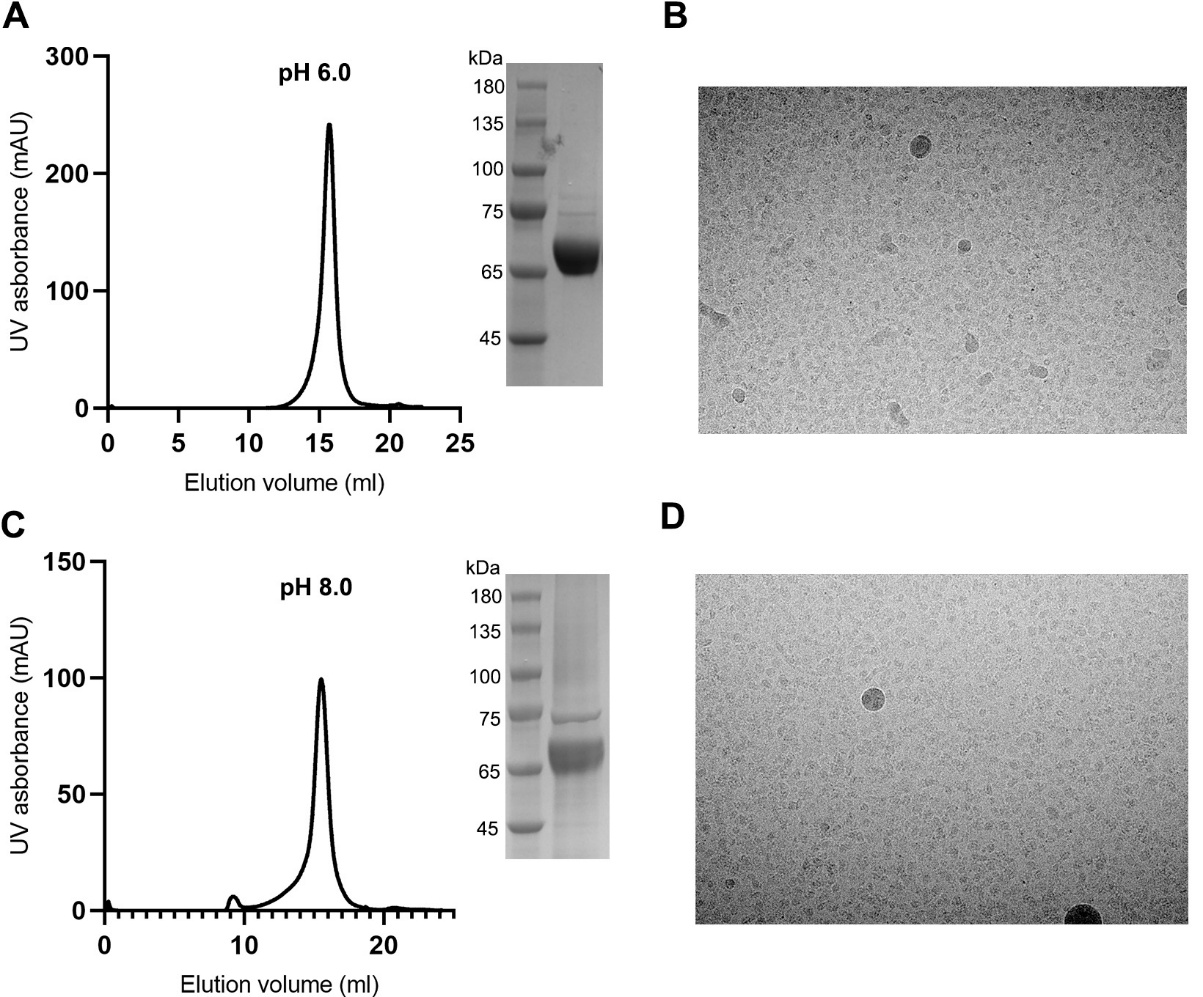


**Fig S1. Purification of AtGTR1. A.** Size-exclusion chromatography of AtGTR1 by Superose 6 (Cytiva) and the protein peak detected by SDS-PAGE gel at pH 6.0. **B.** A representative cryo-EM micrograph of AtGTR1 at pH 6.0. **C.** Size-exclusion chromatography of AtGTR1 by Superose 6 (Cytiva) and the protein peak detected by SDS-PAGE gel at pH 8.0. **D.** A representative cryo-EM micrograph of AtGTR1 at pH 8.0.


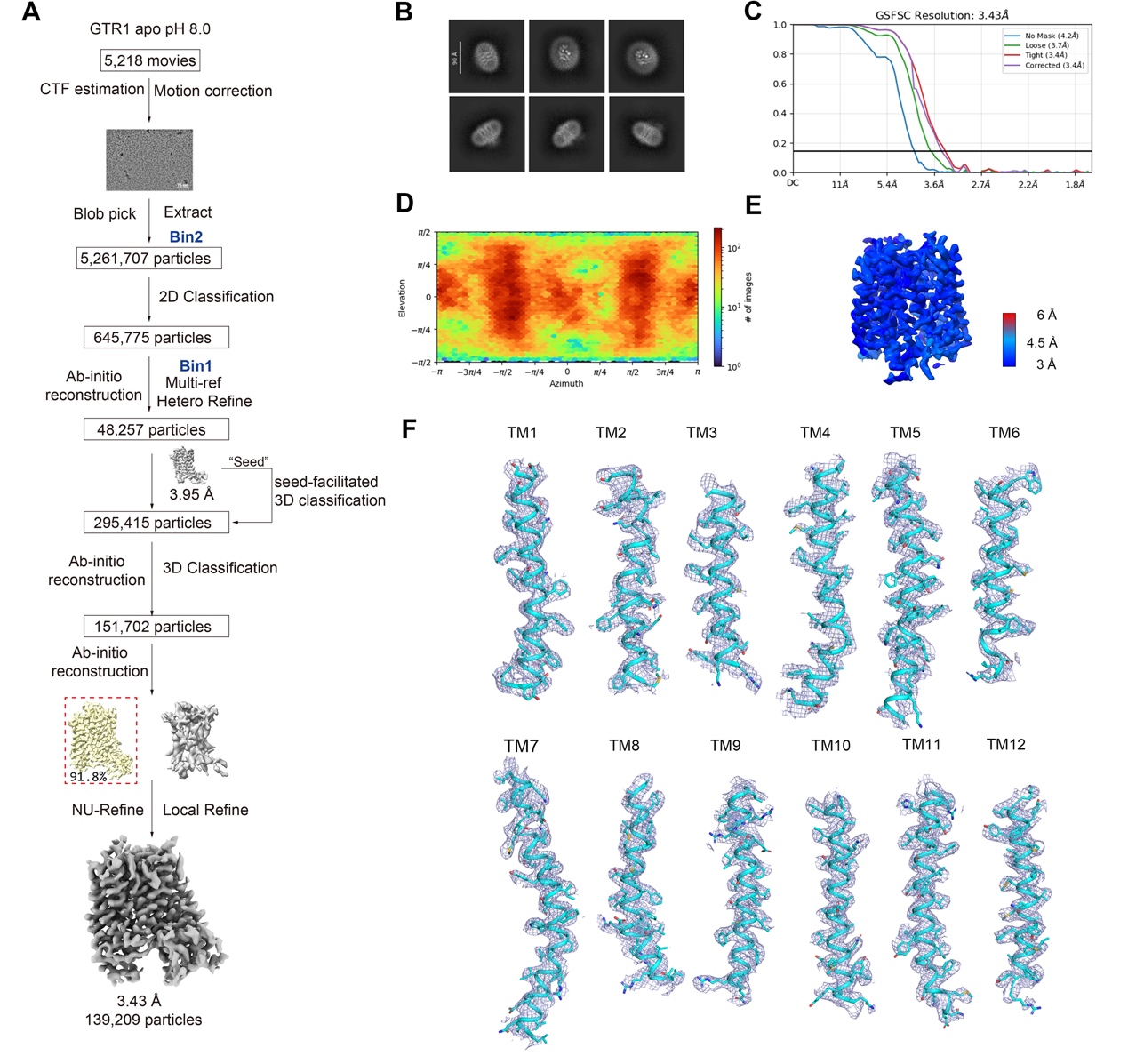


**Fig. S2 | Cryo-EM data processing of apo AtGTR1 at pH 8.0.** **A.** Flowchart of cryo-EM data processing of apo AtGTR1. **B.** Representative 2D class averages. **C.** Fourier shell correlation (FSC) curves between two half maps generated by cryoSPARC. **D.** Angular distribution of particles contributing to the final cryo-EM map of apo AtGTR1. **E.** The local resolution map of apo AtGTR1. **F.** The cryo-EM density maps of all transmembrane helices are shown as mesh (5σ), with atomic models shown as cartoon and sidechains as sticks.


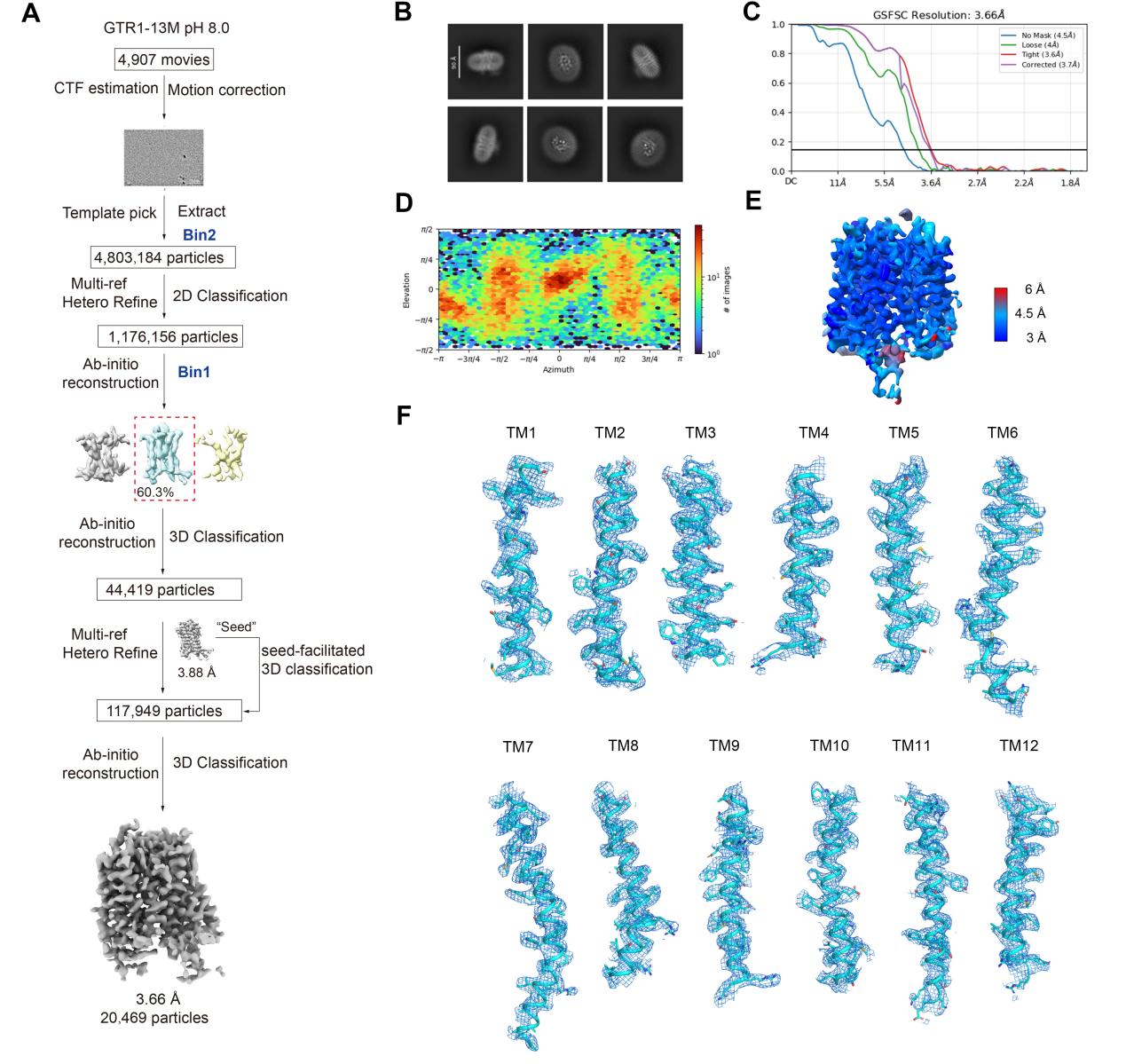


**Fig. S3 | Cryo-EM data processing of AtGTR1-bound I3M at pH 8.0.** **A.** Flowchart of cryo-EM data processing of AtGTR1-I3M. **B.** Representative 2D class averages. **C.** Fourier shell correlation (FSC) curves between two half maps generated by cryoSPARC. **D.** Angular distribution of particles contributing to the final cryo-EM map of AtGTR1-I3M. **E.** The local resolution map of AtGTR1-I3M. **F.** the cryo-EM density maps of all transmembrane helices.


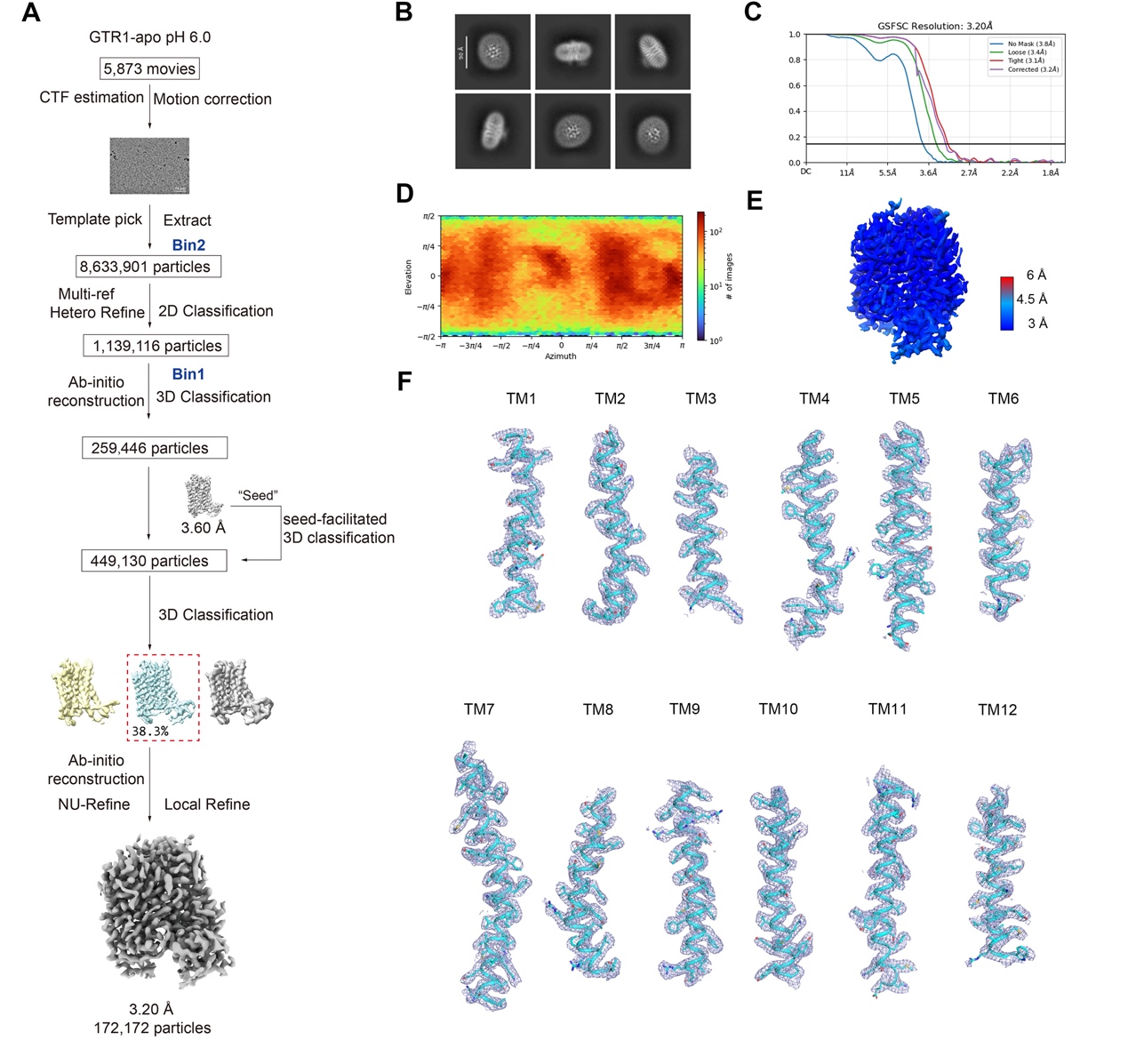


**Fig. S4 | Cryo-EM data processing of apo AtGTR1 at pH 6.0.** **A.** Flowchart of cryo-EM data processing of apo AtGTR1. **B.** Representative 2D class averages. **C.** Fourier shell correlation (FSC) curves between two half maps generated by cryoSPARC. **D.** Angular distribution of particles contributing to the final cryo-EM map. **E.** The local resolution map of apo AtGTR1. **F.** The cryo-EM density maps of all transmembrane helices.


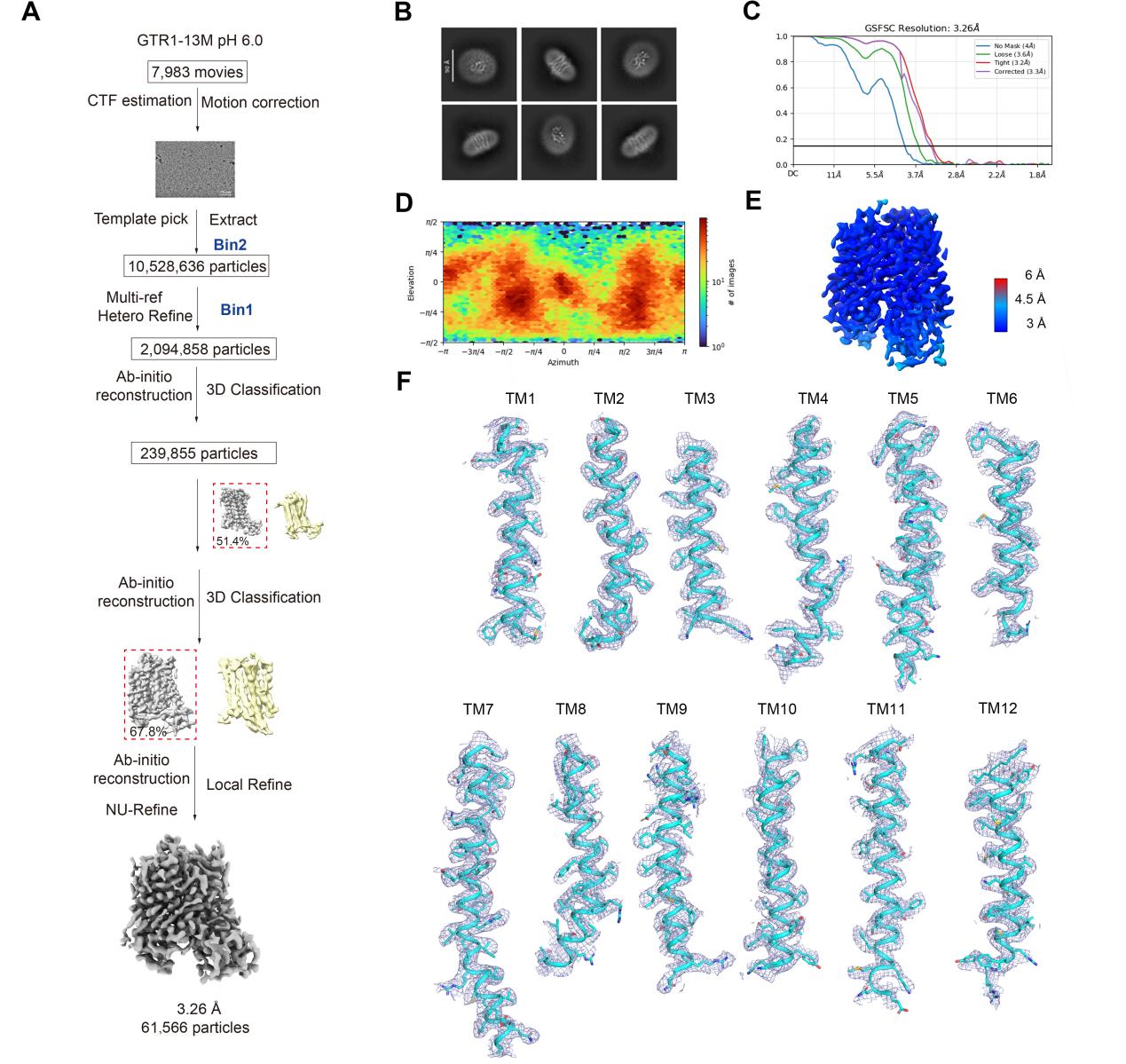


**Fig. S5 | Cryo-EM data processing of AtGTR1-I3M at pH 6.0.** **A.** Flowchart of cryo-EM data processing of AtGTR1-I3M. **B.** Representative 2D class averages. **C.** Fourier shell correlation (FSC) curves between two half maps generated by cryoSPARC. **D.** Angular distribution of particles contributing to the final cryo-EM map of AtGTR1-I3M. **E.** The local resolution map of AtGTR1-I3M. **F.** The cryo-EM density maps of all transmembrane helices.


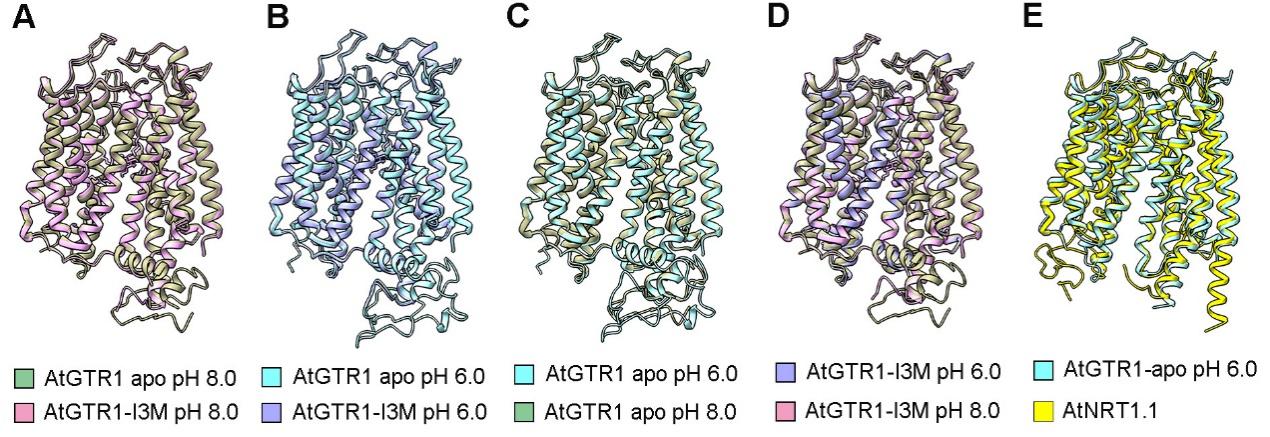


**Fig. S6. Structural comparisons of AtGTR1 at different pH. A.** Superimposition of AtGTR1 at pH 8.0 (apo green, I3M-bound pink). **B.** Superimposition of AtGTR1 at pH 6.0 (apo cyan, I3M-bound magenta). **C.** Superimposition of apo AtGTR1 at pH 6.0 (cyan) and 8.0 (green). **D.** Superimposition of AtGTR1-I3M at pH 6.0 (magenta) and 8.0 (pink). **E.** Superimposition of AtGTR1 apo at pH 6.0 (cyan) with AtNRT1.1 (yellow).


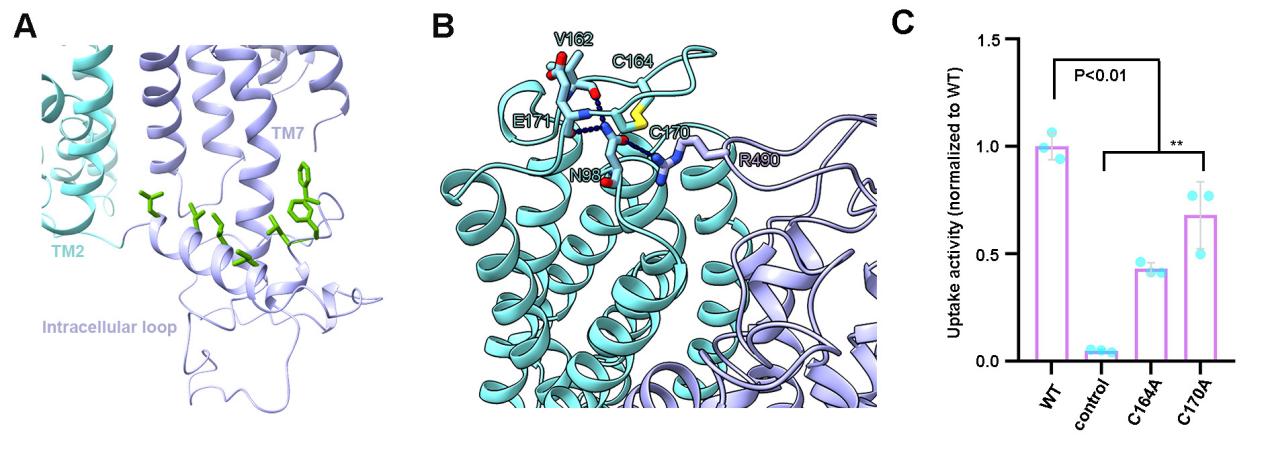


**Fig. S7. Two interactions in AtGTR1-*apo* pH 6.0 state. A.** Hydrophobic amino acid residues in the intracellular loop are shown as green sticks. **B.** N98 interacts with V162, E172, and R490, C164 and C179 form disulfide bond. **C**. Functional validation of AtGTR1 transport activity. Uptake levels are normalized to the wild-type (WT) transporter. Data are presented as mean ± SD from n=3 independent biological replicates. Statistical significance was determined by one-way ANOVA followed by Tukey’s multiple comparisons test (**P<0.01). Individual data points are overlaid as green dots.


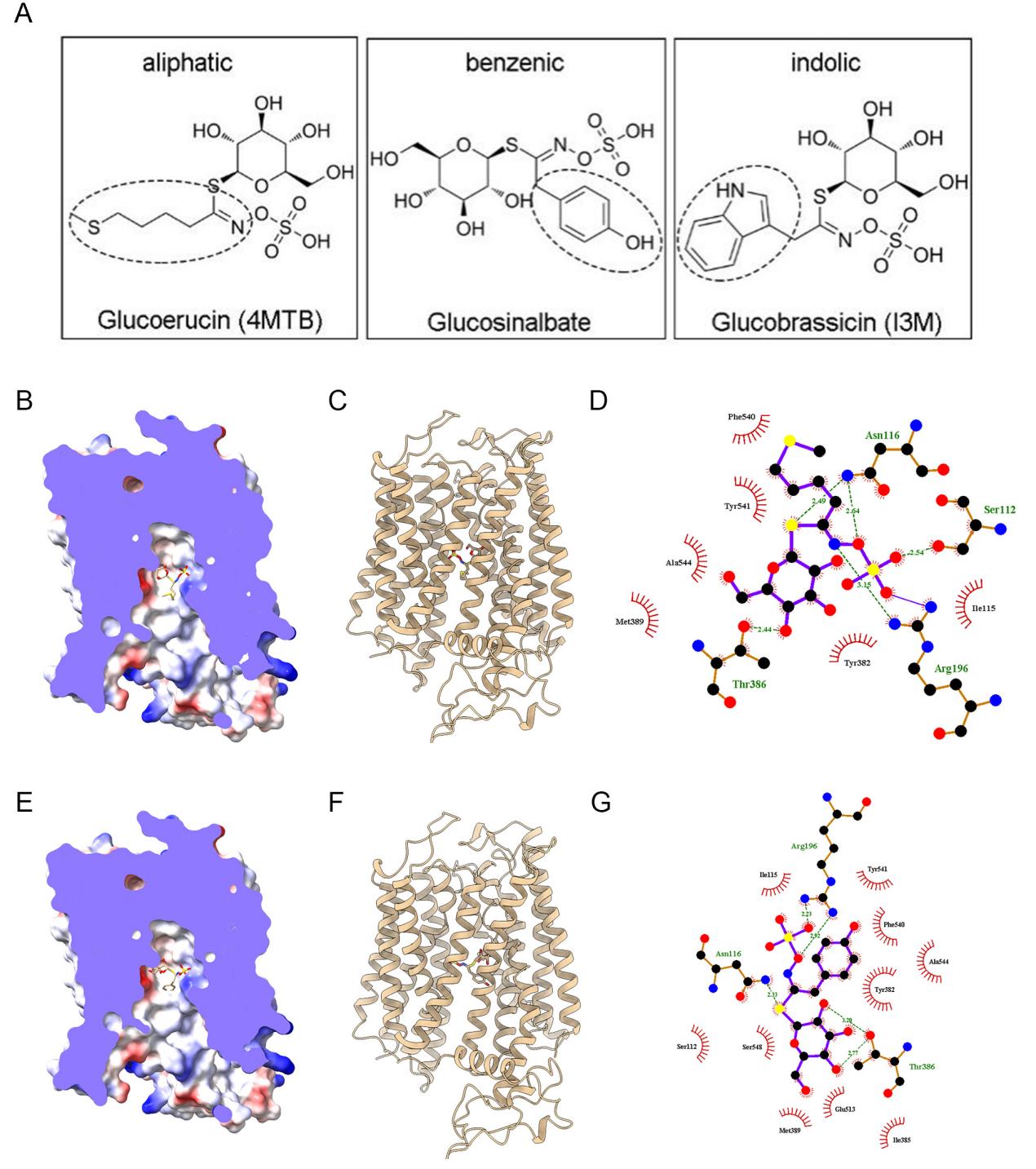


Fig S8. Chemical structures of three representative GLS sidechains and the substrate binding sites of AtGTR1 with 4MTB and Glucosinalbate. **A.** Chemical structures of three representative GLS sidechains. **B.** Slab view of the electrostatic surface of 4MTB binding pocket. **C.** Model of AtGTR1 with 4MTB. **D.** Detailed interaction network between AtGTR1 and 4MTB. **E.** Slab view of the electrostatic surface of 4MTB binding pocket. **F.** Model of AtGTR1 with 4MTB. **G.** Detailed interaction network between AtGTR1 and 4MTB.


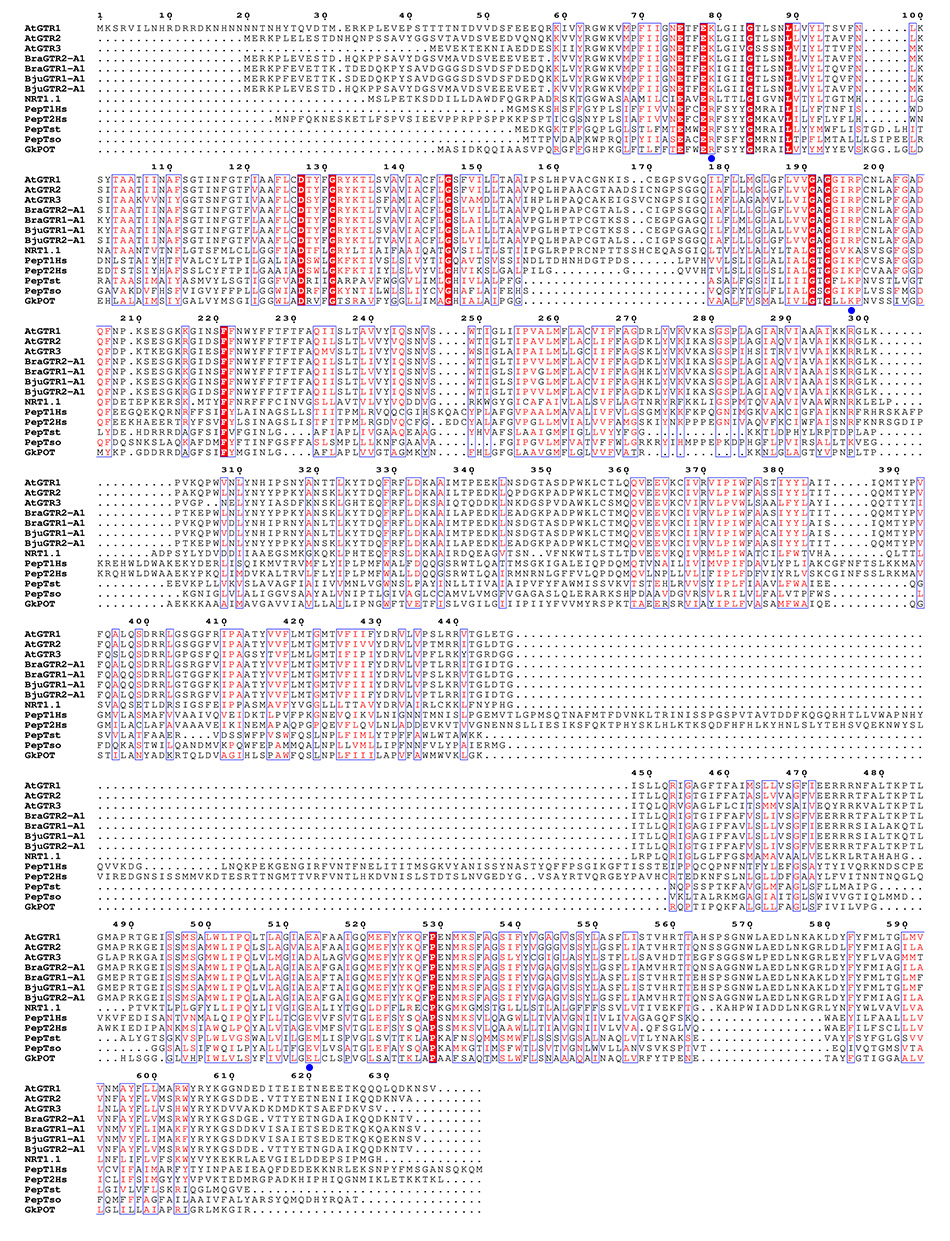


**Fig. S9 Sequence alignment.**

The UniProt IDs for the aligned sequences are: AtGTR1: Q944G5; AtGTR2: Q9LV10; AtGTR3: Q9M9V7; AtNRT1.1: Q05085; PepT1hs: P46059; PepT2hs: Q16348; PepTso: Q8EKT7; PepTst: Q5M4H8; GkPOT: Q5KYD1. BraGTR1-A1: M4DNK9; BraGTR2-A1: M4D0W2; BjuGTR1-A1: BjuA022496; BjuGTR2-A1: BjuA009868. Crucial residues involved in substrate binding in central cavity, K79, R196 and E513, are labeled as blue dot.


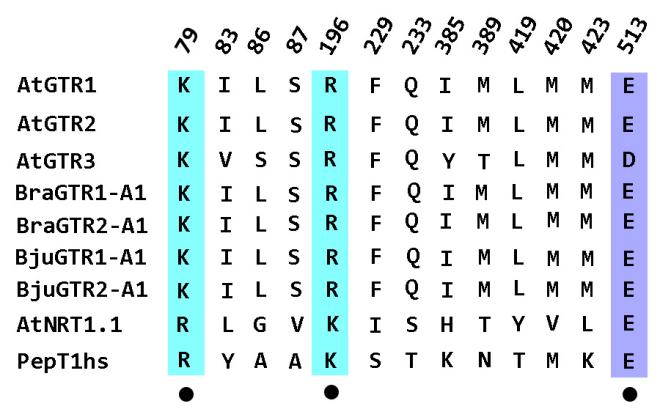


Fig. S10. Sequence alignment of the binding site of AtGTR1 with homologous protein transporters.

**Table S1. Cryo-EM data collection, refinement, and validation statistics**

| ­ | Apo AtGTR1 pH 8.0 | AtGTR1-I3M pH 8.0 | Apo AtGTR1 pH 6.0 | AtGTR1-I3M pH 6.0 |
| --- | --- | --- | --- | --- |
| EMDB code | EMD-68876 | EMD-68875 | EMD-68863 | EMD-68877 |
| PDB code | 23DA | 23CZ | 23CN | 23DB |
| **Data collection and processing** | | | | |
| Microscopy | PKU-IAAS | PKU-IAAS | PKU-IAAS | PKU-IAAS |
| Voltage (kV) | 300 | 300 | 300 | 300 |
| Detector | K3 | K3 | K3 | K3 |
| Magnification | 105,000 | 105,000 | 105,000 | 105,000 |
| Pixel size (Å/pixel) | 0.855 | 0.855 | 0.855 | 0.855 |
| Total electron exposure (e^-^/Å^2^) | 60 | 60 | 60 | 60 |
| Defocus range (μm) | -1.0 to -2.0 | -1.0 to -2.0 | -1.0 to -2.0 | -1.0 to -2.0 |
| Automation software | EPU | EPU | EPU | EPU |
| Symmetry imposed | *C*1 | *C*1 | *C*1 | *C*1 |
| Micrographs collected (no.) | 5,218 | 4,907 | 5,873 | 7,983 |
| Total extracted particles (no.) | 5,261,707 | 4,803,184 | 8,633,901 | 10,528,636 |
| **Reconstruction** |  |  |  |  |
| Refined particles (no.) | 139,209 | 20,469 | 172,172 | 61,566 |
| Data processing software | cryoSPARC | cryoSPARC | cryoSPARC | cryoSPARC |
| Resolution (global, Å) | 3.43 | 3.66 | 3.20 | 3.26 |
| FSC threshold | 0.143 | 0.143 | 0.143 | 0.143 |
| Map sharpening B factor (Å^2^) | -164.6 | -131.9 | -140.6 | -121.9 |
| **Refinement** |  |  |  |  |
| Software | Phenix | Phenix | Phenix | Phenix |
| - real or reciprocal space | Real space | Real space | Real space | Real space |
| Model-Map scores |  |  |  |  |
| - CC | 0.81 | 0.83 | 0.81 | 0.83 |
| No. atoms | 4,027 | 3,686 | 4,246 | 3,732 |
| Protein | 4,027 | 3,638 | 4,246 | 3,684 |
| Ligands | N/A | 48 | N/A | 48 |
| *B* factors (Å^2^)  Protein  Ligands | 194.91  N/A | 171.90  221.90 | 50.05  N/A | 124.76  156.33 |
| R.m.s. deviations |  |  |  |  |
| Bond lengths (Å) | 0.004 | 0.006 | 0.004 | 0.009 |
| Bond angles (°) | 0.737 | 1.029 | 0.58 | 1.266 |
| **Validation** |  |  |  |  |
| MolProbity score | 1.53 | 1.68 | 1.94 | 1.69 |
| Clashscore | 4.00 | 7.95 | 5.06 | 8.92 |
| Poor rotamers (%) | 0.00 | 0.00 | 0.00 | 1.00 |
| Ramachandran plot  Favored (%)  Allowed (%)  Outlier (%) | 95.20  4.80  0.00 | 96.34  3.66  0.00 | 99.45  0.55  0.00 | 97.00  3.00  0.00 |
